# The Patient's View of Nursing Care after Hip Fracture

**DOI:** 10.5402/2012/863291

**Published:** 2012-07-03

**Authors:** Ami Hommel, Marie-Louise Kock, Jeanette Persson, Elisabeth Werntoft

**Affiliations:** ^1^Department of Orthopaedic, Lund University Hospital, 22185 Lund, Sweden; ^2^Department of Health Sciences, Lund University, 22100 Lund, Sweden

## Abstract

*Background*. The pathway for patients with a hip fracture described in this study is a fast track. Many studies have focused on prevention of various complications but, so far, the patient's view of nursing care has not been highlighted. *Aim*. The aim of the study is to illuminate the patient's view on nursing care when treated for a hip fracture. *Method*. Ten patients were interviewed. A content analysis design was conducted. *Findings*. From the analysis, four main categories emerged: *waiting times; pain/pain relief and mobilisation; attitude/information and sense of security; complications*. *Conclusion*. Patients generally felt satisfied with the nursing provided. The staff created a feeling of security and showed interest and empathy for the patient. However, patients experienced a stressful waiting for surgery, and patients who developed confusion waited more than 24 hours for surgery. Therefore, waiting time must be decreased. Furthermore, patients' descriptions of a variety of pain problem show, for example, that good collaboration between the nurse and physiotherapist is critical for achieving good pain relief before mobilisation. Nursing staff need to be attentive and should elicit the patient's feelings through patient-focused communication in order to relieve anxiety about going home.

## 1. Introduction

Traditionally, the focus of outcome measurement for patients with a hip fracture has been on mortality and surgical implant success. Increasing recognition of the need to diversify outcome measurements has led to the creation and use of a number of outcome scales, for example, general quality of life, activities of daily living, mobility and physical performance; and hip-specific [1]. Many studies have focused on early mobilisation and prevention of various complications [2–4] but, so far, the patient's view of nursing care has not been highlighted.

Globally, the number of patients per year with a hip fracture has been forecast to increase from 1.66 million in 1990 to 6.26 million by the year 2050 [5, 6]. The highest incidence is seen in Scandinavian countries [7, 8]. Hip fractures constitute a serious and common health problem among older adults from both the individual and the public health perspectives, due to the age and comorbidities of the affected patients [9–11]. The worldwide cost of hip fracture treatment including rehabilitation is expected to rise from US$ 34,800 billion in 1990 to US$ 131,500 billion in 2050 as the number of older people increases in coming decades [12]. Therefore, different integrated care pathways have been adopted around the world [13, 14] to decrease the burden on the healthcare system and to optimise the treatment to minimise the patients' suffering as far as possible throughout the pathway [4]. In an attempt to further optimise the state of the patients with a hip fracture, a new clinical pathway was implemented at the Department of Orthopaedics, Lund University Hospital, Sweden, in 2007. This includes a rapid transition with higher priority level by SOS alarm. Besides giving patients oxygen, pain relief, and fluids in the ambulance—which was introduced in 2003—blood tests and ECGs are also taken. In 2003, a pathway was introduced in which the patient was transported first to the acute & emergency room (A&E), and then directly to the ward after X-ray. In this new pathway—introduced in 2007—the ambulance crew transports the patient, after consulting a physician, directly to the X-ray unit and then to the ward, thus, avoiding the patient having to be admitted to A&E. As before, mobilisation takes place one day after surgery. All patients are offered nutritional drinks twice a day. The aim of the study was to illuminate the patient's view on nursing care when treated for a hip fracture.

## 2. Method

A qualitative content analysis design was conducted, using a semistructured interview guide influenced by Burnard [15].

 A convenience sample with 10 patients was recruited from two orthopaedic wards at the Lund University Hospital, Sweden. The inclusion criteria were as follows: hospitalised for hip fracture, proficiency in the Swedish language, admitted to the hospital through the new pathway, and completion with a positive outcome of a cognitive function test, the Short Portable Mental Status Questionnaire (SPMSQ) [16] consisting of a questionnaire with ten general questions. Eight to ten correct answers indicate intact cognitive function, describing the patient's memory capacity, thought structure, and orientation [17]. This test was conducted by a nurse on the orthopaedic wards. Two of the authors (M. L. Kock and J. Persson) regularly visited the wards and approached patients eligible for the study and gave them written information about the study. If a patient wanted to participate in the study, a suitable appointment was made for the day or days before discharge. Patients were given written information and a consent form so that they could contact the authors if they had any questions. One patient called to cancel because of fatigue. All other patients gave their informed consent to participate in the study before the interview took place.

 All interviews were conducted by MLK and JP in a separate room on the orthopaedic ward and were documented by a tape recorder and supporting notes. The interviews were transcribed verbatim. The interview commenced with an open question, *“Would you like to tell us about the day you fell?”* in order to get the patient talking in an open and relaxed manner. This was followed by those questions in the interview guide that had not been answered in the opening question. The interview concluded with the question, *“Would you like to tell us how you feel now as you look back on your hospital stay?”*, the objective being for patients to take the time to consider whether there was anything they had left out and wanted to convey.

The interviews were first analysed by MLK and JP using Burnard's content analysis, which consists of 14 separate steps. A manifest content analysis was used in order to find expressions and shared patterns that were central to the patients' statements. The interview material was read repeatedly after each interview to achieve understanding of the informant's life world (Burnard 1991). Open coding was performed. The text was divided into similar phrases. Each of the authors then separately created different categories, which resulted in 15 categories. These were compared and similar categories collapsed into four groups. The interview printouts were reread alongside the developed category list, while colour markings were entered in the printouts to ensure that all aspects of the interviews had been included.

 To increase trustworthiness, the results were continuously discussed among the authors and EW developed categories that were compared with those of M. L. Kock and J. Persson. The categories were found to agree well. Four main categories emerged and were studied in order to create a text containing the most appropriate quotations in each category.

## 3. Ethical Considerations

The study was approved by the Research Committee for Ethics in Health Education at the Lund University. The study was conducted according to the Declaration of Helsinki, 1964. The informants were given oral and written information that the study was voluntarily and that they had the right to withdraw their participation at any time without any explanation.

## 4. Results

Nine women and one man participated in the study; mean age was 78 years. External factors were a major contributing reason why the patients fell and sustained their hip fracture. From the analysis, four main categories emerged: *waiting times; pain/pain relief and mobilisation; attitude/information and sense of security; complications.* The participants were numbered (1–10) and the number after each quotation shows who was talking.

### 4.1. Waiting Times

The patients reported that the ambulance arrived quickly, within 15–20 minutes. The ambulance crew told them that a project was ongoing so they would not have to be admitted into A&E but directly to X-ray and then to the ward

*I was afraid of sitting there for hours, being in pain (3).*

The patients expressed positive reactions to the fact that all tests and examinations took place in the ambulance in order to speed up processing in the hospital. A few patients reported having to make a short stopover in A&E because there had been a major accident and the physician was not available for consultation. The majority of patients reports that there was no waiting time for transportation to and from the X-ray unit. They describe their waiting time as very short and that they were taken to the ward directly afterwards. Most patients described the waiting time for surgery as lengthy. It became clear that the patients lost their perception of time and place. They also felt very anxious about the pain and they worried that the waiting time could contribute to infections

*Waiting for surgery was extremely stressful; you did n't really know where you were, or whether it was day or night (8).*

*It took more than 24 hours/…/you do n't know whether infections can occur, that can happen, of course/…/then I had to wait some more; that was bad luck! (4).*

The interviews showed that the patients believed they would have surgery on the same day they arrived at hospital. When this did not happen, the long waiting time made them dejected and upset. The patients perceived the waiting time to be lengthy, but when it was time for surgery, they were not mentally prepared because everything happened very quickly

*… and then the next day I expected that I would have to wait because they couldn't promise when there would be a surgery, and then suddenly they said that it was time, and then I wasn't mentally ready (8).*

While waiting for surgery, patients also reported feeling hunger, thirst and having a dry mouth

*… I found it stressful to wait; I had to wait all day, my mouth was dry and I wasn't allowed to drink (5).*

Patients who underwent surgery the same day, after waiting a few hours on the ward, had no perception of waiting time

*No, no it did n't take long, it just moved right along, it really did. It was great (3).*

### 4.2. Pain/Pain Relief and Mobilisation

It emerged from the patients' narratives that they felt discomfort from pain and that this appeared in different ways such as intense or stabbing pain, radiating pain down toward the groin, numbness of the leg, and pain in the hip. These expressions recurred when the patients described their pain

*It does n't ache, it's intense pain, the pain is n't ordinary, it's intense and there is a difference (10).*

Patients perceived the hip pain to be worst in conjunction with movement; when they laid still, the pain was nonexistent except at the start of the hospital stay when the pain was constant. The pain was also described as very severe when rising from the bed. Bathroom visits, with an unsuitable sitting position, were also considered an ordeal. One informant suggested that this was because the toilet booster seat is not adapted for this type of injury. The patients reported that the pain they experienced gradually decreased during the hospital stay.

Although the patients found the pain relief sufficient, they still claimed to be in great pain. They spoke about fear of adverse effects and that they should get too much medicine although they had trust in the staff's knowledge about what medicine they were offered. The patients explained their acceptance of pain by saying that they, after all, have sustained a major trauma and that they simply have to expect it to be painful. The patients said that since they were constantly being given medicine, this ought to be sufficient for the pain. The Visual Analogue Scale (VAS) pain instrument used by the staff was perceived as difficult to rate

*… they have demonstrated that pain scale to me, but the rating is n't that easy, it is n't just like using a thermometer where there is an exact scale. At first I think I probably had 4-5, then I thought I might have 6, so it is n't that easy (4).*

Patients found mobilisation to be brusque in the first few days, but said that the staff did what they have to and in the best possible way

*I screamed like a madman; everyone did. I could hear what stage they were at (10).*

Patients felt tense and scared during mobilisation and were afraid of falling again. They described their legs as stiff, numb, and hard to raise and it was difficult to get up and walk. Patients felt that they needed more training, preferably with a physiotherapist

*I had to get up and stand and started with two steps. Then back to bed again, but starting yesterday, I was sitting in my room and thought, well there it stands (the walking frame), and there is the window sill, I simply have to get to it by myself and go to the bathroom, and I managed. Gold star! (1).*

### 4.3. Attitude/Information and Sense of Security

“Wonderful” and “helpful” were recurring concepts when attitude came up in the patients' statements. The ambulance crew was lauded and perceived as highly professional. Patients found the alarm bell in the room made them feels secure, calling it a quick and secure way of summoning the staff not only when help was needed, but also when questions and concerns arose. The patients talked about the staff coming up with training tips, ideas, and suggestions on how they should do to achieve the best results and get well again. It is clear that they felt well-looked after by the staff

*… you get help with everything, as soon as you ring the bell they come/…/offer tips on how to train the leg, it makes you feel secure (6).*

*They are all so friendly and kind and nice. I think the staff here are tops, and their patience is incredible; they deserve a medal (4).*

Patients reported that they were satisfied with the information about their hip, the surgery, and the training of the leg. They mentioned having received verbal information and having understood it. There was a weekly information session on the ward about hip fractures, which was positively received by the patients who experienced the session as fruitful. Written information was not provided to the same extent

*… you gave me the first brochure, and I was pleased to get it (7).*

Anxiety about getting home was a recurring theme by the patients. Many questions and concerns arose about their future. Patients described themselves as being in need of assistance at home since their situation will be different from before as they had no help at home. Above all, they need help with activities such as showering, physiotherapy, and transportation to and from their home. They also voiced their concern about family members that perhaps will not have time to help them when they return home, which made them feel insecure and uneasy

*… I have to get dressed every day, manage my ablutions and everything/…/I do have a sister-in-law who perhaps can help me/…/but they have so much on their hands with the children and all (5).*

The room atmosphere was described as good by the patients. They said that their roommates were patient and that all understood and showed respect for each other's habits, since they all were ill and in the same situation.

## 5. Complications

It emerged from the interviews that hallucinations had occurred and that these, according to the patients, were caused by excessive pain relief. The hallucinations occurred at night but disappeared in the morning. Several patients also described seeing “silent old men” everywhere, on the curtain and on the chairs in the room. They tried to speak to the old man, but got no response

*… in the night I saw shadows, it was like no faces, and they stood around me, they were figures, nobody made a sound, no faces that I can remember, it was very strange (4).*

*… then I thought a man was standing there/…/he was so close that I thrust out my hand*

*/…/and my hand just vanished out in empty space/…/so I was hallucinating (9).*

It emerged that when the patients told the staff about their hallucinations, the staff explained that this happens and was not uncommon, and that it could be caused by the pain relief medicine, but also by their current situation, which involved a new environment and their surgery. The patients found this information to have a calming effect.

It also appeared that the patients had sleeping problems early in their hospital stay, relating primarily to pain and difficulty changing position; toward the end of the hospital stay they found themselves sleeping better. The patients also suggested that the sleeping problems were caused by anxiety about getting home, strange noises, snoring, and by resting too much during the day, which made it difficult to sleep in the night. A fear of taking sleeping pills also emerged, which they suggested was a reason for their sleeping problems

*… I try to sleep, it is n't good to get used to sleeping pills, then you might become dependent; that's something you should try to avoid. It's narcotics, after all (2).*

The patients' responses showed that none developed pressure ulcers, nor did anyone have a wound infection. Problems that were experienced were stated as swelling of the leg, low blood count, and constipation

*My leg is swollen and it's very numb/…/My blood count has been low and they've given me blood. So that has made me tired (8).*

Patients considered the food to be good but found that they had no appetite. They found the portions too large and they were not used to have cooked meals twice daily. They suggested that one cooked meal per day is sufficient, but regarded it as positive having different dishes to choose from. However, the patients indicated that they mostly had their meals in their room because of difficulty getting to the dining room

*… what is great is that you can choose what you want to eat/…/being able to choose so that the food suits everyone (8).*

The result suggested that nutrition drinks are not served regularly. Patients who received nutrition drinks did not like the taste and stated that they were not told why they should drink them. Patients who did not receive nutrition drinks wondered what they were

* No, no, I haven't been given nutrition drinks, not yet in any case, one cann't tell what will happen. Is that something I ought to have tried, girls? Have I missed something? (7).*

## 6. Discussion

The purpose of this study was to describe the patients' view of nursing care when they were treated for a hip fracture. The findings show four main categories emerge: *waiting times; pain/pain relief and mobilisation; attitude/information and sense of security; complications*.

In summary, patients did not experience significant waiting times for the ambulance, X-rays, or transfer to the ward. The delay came at a later stage in the wait for surgery. Most of the patients had to wait more than 24 hours for surgery and those who developed confusion were found in this group. A long waiting time for surgery increases the risk of confusion [18]. A long waiting time for surgery can also lead to pressure ulcers [19]. No patients in the study developed pressure ulcers despite most having waited more than 24 hours for surgery; this could be because the personnel is trained in the prevention of pressure ulcers, but also because of the active role they have in the patient's early mobilisation after surgery and because the new pathway, with its faster overall processing, has influenced the outcome. Patients' descriptions of the stressful experience of waiting for surgery may be a reason to investigate and address the causes of the delay.

Descriptions of a dry mouth and thirst were also seen as a problem while waiting for surgery. Fasting and the dry hospital air cause the patient to suffer thirst and a dry mouth. It is most important to practice oral hygiene as often as necessary and to know the guidelines for oral intake before surgery so that the patient does not suffer needlessly.

Several studies have shown that pain treatment in this patient group is often inadequate [20–22]. It emerged from this study that the patients experienced much pain especially in conjunction with mobilisation, in particular when rising from the bed and when visiting the bathroom. Hallström et al. [23]; found that patients avoided moving both before and after surgery because of pain, a fact which made mobilisation more difficult. This shows that it is important to provide pain relief before mobilisation and that clear communication between the nurse and physiotherapist is critical for ensuring that pain relief is given in good time before the training. It also appeared that patients accepted being in pain because they had undergone major surgery. This has also been showed by Dahlman et al. [24] and sHallström et al. [23] while the patients accepted having pain after major surgery, they also did not want to trouble the nurse to ask for pain relief. Nurses had different ways of communicating with patients [25]. Those nurses who asked more direct questions about the patient's pain also received clearer answers, and the pain could be more effectively addressed. When clear questions were not made, it was easier to misinterpret the patient's answer about the pain experienced, and pain relief was not provided. For the nurse, it is important to be attentive to the patient's body language and facial expression. Ageing leads to changes in the body's metabolism and elimination of drugs which in turn increases the risk of adverse effects. Patients relied on the staff's knowledge of tablets that were offered and felt that they had received sufficient pain relief, although they were still in pain [23]. Fear of taking too many tablets was also expressed. The patients' earlier experiences of adverse drug effects, in the form of hallucinations, caused them to forego pain relief because of fear. Instead, they tried to conceal their pain, which in turn led to an exacerbated pain experience and impaired mobility. Nurses who participated in an education day on pain treatment gave a higher dose of intravenous pain relief than previously [24] which led to the patients experiencing less pain. Education days on the action of drugs—pharmacological as well as physiological—should be recommended for nurses. This would allow patients with a fear of tablets to be informed by the nurse about the effect of the drug and the negative effect of pain on the body. There should also be a strong emphasis on individual pain relief and monitoring in order to reduce the risk of adverse effects.

Results showed that patients felt well looked after and secure in the hospital. The staff's attitude was “fabulous”, as was their patience and helpfulness, but several patients, nevertheless, felt anxious about going home. The patients had come to the hospital straight from home and were used to coping on their own. On returning home, they would need support and assistance, both from the local authorities and their family. The patients would be faced with an entirely new situation where they are dependent on others. Maslow's hierarchy of needs makes it clear that not only primary needs such as food and drink have to be met, but that secondary needs and meta needs, such as a sense of security and the meaning of life, also have to be more clearly addressed during illness. All patients in our study lived at home, which may be a contributing reason why the result showed that many were anxious about going home. It is important for nursing staff to interpret signals from the patients and try to elicit their feelings as well as possible; this allows the staff to provide information and respond to concerns in order to relieve uncertainty and make patients feel less insecure about going home. During care planning, nursing staff with the best knowledge of the patient should attend.

The study shows that patients appreciated the ability to choose between different dishes, but at the same time had no appetite. They stated that portions were too large and that they mostly took their meals in their room. The focus should be on individual nursing care of the patient with respect to nutrition; food intake and support around mealtimes; information and the personal involvement of patients in increasing their food intake. A change of environment at mealtimes could have contributed to improving the patients' appetite. It would get them out of their room for a while into the dining room where they would meet other patients with whom they could share a meal and communicate. Since the lack of appetite was a recurring problem, nurses must be on the lookout for patients whose nutritional status is impaired or who are in the risk zone so that nursing measures can be taken as quickly as possible. The reason why the staff did not offer nutrition drinks or serve them regularly may have been that the patients were in a good nutritional state. This may also have been a reason why no patient developed pressure ulcers, as the staff's active role with respect to the patient's choice of dishes contributed to the patient's involvement.

This study has limitations; 10 patients were included, nine women, and only one man. The distribution in Sweden is 70% women, so, it would be preferable to have included more men in the study, which might have given other results, as well as a higher number of included patients. Mean age in Sweden among patients with a hip fracture is 83 years. In this study, mean age was 78 years. If the study group had been older, we might have had other results since older people are more prone to suffer from complications. However, we hope that the result presented in this study highlights the patients' view, leading to further improvements of the nursing care of these frail patients.

## 7. Conclusion

Our perception is that patients generally felt satisfied with the nursing provided throughout the clinical pathway and that this to a large extent was due to the manner in which the staff created a feeling of security and showed interest and empathy for the patient. The study demonstrates that patients with hip fracture have a major need of nursing care. The patients' experiences of long waiting times for surgery should lead to investigation and attempts to remedy the causes of delay. For a nurse, it is of major importance to interpret the patient's expression of pain, and education days on pain treatment for nurses are desirable. Good collaboration between the nurse and physiotherapist is critical for achieving good pain relief before mobilisation. Nursing staff need to be attentive and should elicit the patient's feelings through patient-focused communication in order to relieve anxiety about going home. We believe, therefore, that measures such as these can lead to an improvement in nursing care from the patient's perspective.
